# The expanding toolkit for structural biology: synchrotrons, X-ray lasers and cryoEM

**DOI:** 10.1107/S2052252519002422

**Published:** 2019-03-01

**Authors:** Stephen P. Muench, Svetlana V. Antonyuk, S. Samar Hasnain

**Affiliations:** a School of Biomedical Sciences and Astbury Centre for Structural Molecular Biology, University of Leeds, Leeds LS2 9JT, England; b Molecular Biophysics Group, Institute of Integrative Biology, Faculty of Health and Life Sciences, University of Liverpool, Liverpool L69 7ZX, England

**Keywords:** synchrotron crystallography, serial femtosecond crystallography, cryoEM, MSOX

## Abstract

Structural biology continues to benefit from an expanding toolkit, which is helping to gain unprecedented insight into the assembly and organization of multi-protein machineries, enzyme mechanisms and ligand/inhibitor binding. The combination of results from X-ray free-electron lasers (XFELs), modern synchrotron crystallographic beamlines and cryo-electron microscopy (cryoEM) is proving to be particularly powerful. The highly brilliant undulator beamlines at modern synchrotron facilities have empowered the crystallographic revolution of high-throughput structure determination at high resolution. The brilliance of the X-rays at these crystallographic beamlines has enabled this to be achieved using microcrystals, but at the expense of an increased absorbed X-ray dose and a consequent vulnerability to radiation-induced changes. The advent of serial femtosecond crystallography (SFX) with X-ray free-electron lasers provides a new opportunity in which damage-free structures can be obtained from much smaller crystals (2 µm) and more complex macromolecules, including membrane proteins and multi-protein complexes. For redox enzymes, SFX provides a unique opportunity by providing damage-free structures at both cryogenic and ambient temperatures. The promise of being able to visualize macromolecular structures and complexes at high resolution without the need for crystals using X-rays has remained a dream, but recent technological advancements in cryoEM have made this come true and hardly a month goes by when the structure of a new/novel macromolecular assembly is not revealed. The uniqueness of cryoEM in providing structural information for multi-protein complexes, particularly membrane proteins, has been demonstrated by examples such as respirasomes. The synergistic use of cryoEM and crystallography in lead-compound optimization is highlighted by the example of the visualization of antimalarial compounds in cytochrome *bc*_1_. In this short review, using some recent examples including our own work, we share the excitement of these powerful structural biology methods.

## Abstract 2

The expanding toolkit of structural biology, with further advances in the technologies associated with cryoEM, synchrotrons and XFELs, and the ease of their use, should continue to enable the wider community to address more complicated and demanding scientific questions, ensuring the ‘pole position’ of structural biology.

## 1. Introduction   

The ability to visualize atomic details in three dimensions has proven transformative in the way that we think of biological machines and understand the chemistry that is performed in a complex, coordinated manner at, for example, the catalytic core of an enzyme or protein complex such as photosystem II. Until recently, X-ray crystallography and nuclear magnetic resonance (NMR) spectroscopy have been the main players. NMR does not require protein molecules to be coaxed into crystals, but carries a limitation on the size of the molecule for which a structure can be determined. The tremendous success of X-ray crystallography over the last 30 years has largely been owing to (i) the availability of increasingly brighter synchrotron X-ray sources, (ii) the ability to screen crystallization conditions using robotics and *in situ* visualization platforms, (iii) the use of highly optimized beamlines with efficient single-photon-counting detectors, (iv) automation, (v) optimized user-friendly data-acquisition systems and (vi) sophisticated data-processing/analysis packages. The drive towards ever-increasing brightness of X-ray sources has led to diffraction-limited synchrotron-radiation (SR) sources (Eriksson *et al.*, 2014 ▸; MAX-IV in Sweden and SIRIUS in Brazil) and the establishment of X-ray free-electron laser (XFEL) facilities in the USA, Japan, Germany, Switzerland and South Korea. XFELs provide huge gains in peak intensity, allowing high-resolution molecular structures to be obtained from crystals of a few nanometres in size. They enable the structure determination of complex systems both at room temperature and at cryogenic temperature without any radiation damage, as the X-ray pulses are shorter than the vibrational/rotational frequencies. In a way, these are ‘time-frozen’ structures and provide a true representation of the molecule prior to any effect from X-ray-induced photochemistry. Despite the success of crystallo­graphy, a serious bottleneck remains, namely the ability to obtain diffracting crystals. This is particularly the case for membrane proteins, large multi-protein complexes and encounter complexes, which by definition are transient in nature, but are crucial for understanding biological function. In the context of cryoEM, the revolution that we are witnessing was aptly described as the ‘method of the decade’ at a micro-symposium at the 24th IUCr Congress in August 2017 (Hasnain, 2016 ▸). CryoEM has already provided high-resolution (<2 Å) structures of multi-protein complexes with structural details comparable to those of crystal structures. In 2017 alone, the number of structures determined by this method exceeded the number that had been determined prior to 2014. This was the year when crystallography marked the 100th anniversary of its first Nobel prize through the United Nations’ declaration of 2014 as the International Year of Crystallography.

**Figure 1:**
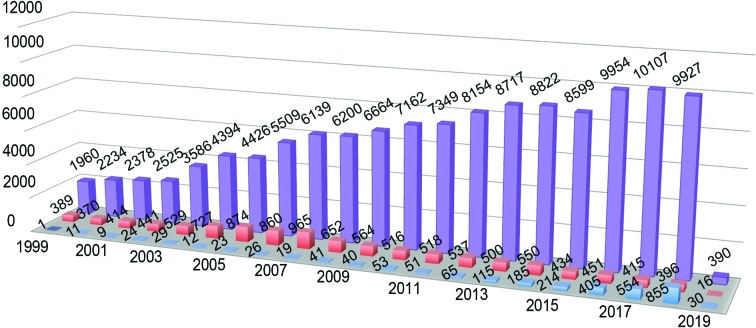
The numbers of structures deposited in the Protein Data Bank (PDB; Berman *et al.*, 2000 ▸) in a particular year associated with the X-ray crystallography (lilac), NMR (salmon) and cryoEM (blue) methods. Note that the data for 2019 are only to 17th January.

There are currently 148 037 structures (as of 17th January 2019) in the Protein Data Bank (PDB), with ∼90% being determined by X-ray crystallography, of which 82% were obtained using synchrotron beamlines. Fig. 1 ▸ provides a detailed breakdown of structures determined by X-ray crystallography, NMR and EM. The pace of change for cryoEM came at the turn of the century, when 11 structures were deposited in 2000. We now have ∼2800 deposited structures that were determined by cryoEM. For NMR, structure deposition peaked in 2007, and it is currently producing ∼400 structures per annum. It is proving to be a powerful approach to study inherently disordered proteins and for rapidly screening fragment libraries. X-ray crystallography still dominates the field, and has now reached ten thousand structures per annum. Despite the wealth of structures in the Protein Data Bank, a closer examination reveals that 89% of the structures, *i.e.* 126 994, are of proteins or complexes with a molecular weight of less than 160 kDa. Furthermore, only 4% of the deposited structures have a molecular weight in excess of 300 kDa. This deficiency is largely owing to the limitations of obtaining high-quality protein and the unpredictability of obtaining diffraction-quality crystals. Given that cryoEM comes into its own for proteins with molecular weights in excess of 120 kDa, one may expect this structural gap to be overcome in the coming decade.

## 2. Damage-free structures using X-rays: a unique place for XFELs   

The highly intense microfocus crystallographic beamlines equipped with efficient photon-counting detectors at modern synchrotrons have increased the quality and the number of structures that can be obtained. Much smaller crystals (20–50 µm) can be used for high-resolution crystallographic data collection. However, this has come at the cost of an increased absorbed X-ray dose and consequent radiation-induced changes that can occur during data collection (Hough *et al.*, 2008 ▸). Neutron crystallography has for decades remained the only radiation-damage-free structural probe, but the advent of femtosecond XFEL crystallography provides a new opportunity. Owing to the time structure of the pulse, damage-free structures can be obtained using XFELs from much smaller crystals and from more complex macromolecules, including membrane proteins and multi-protein complexes, as illustrated by the following examples.

### 2.1. Photosystem II   

**Figure 2:**
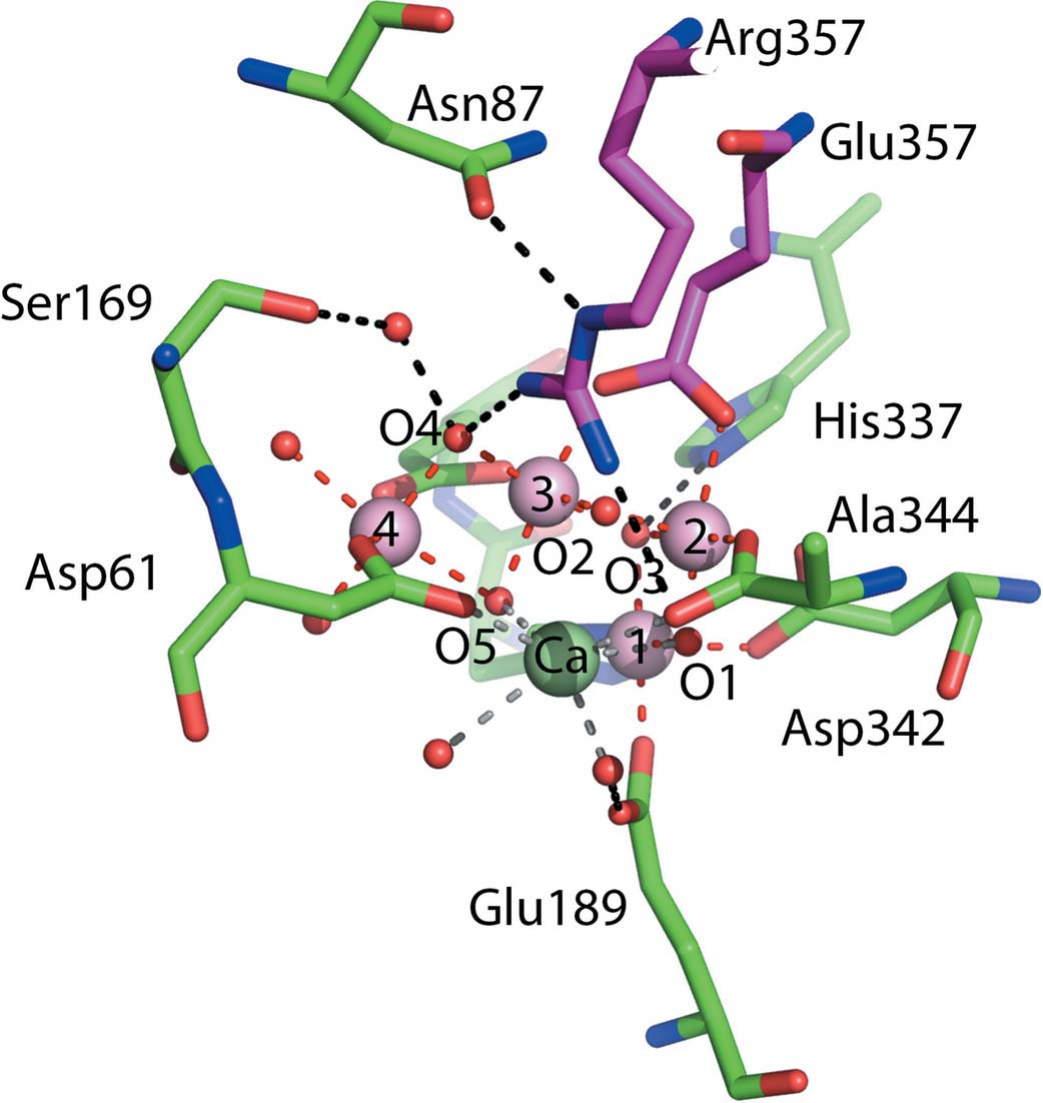
The catalytic centre of the native XFEL-determined structure of photosystem II (two-flash data set; PDB entry 5gti) showing details of the CaMn_4_O_5_ cluster and the surrounding residues assigned at 2.35 Å resolution. Coordination bonds are shown in red and hydrogen bonds in black; residues are colour-coded according to the chain identifier. Manganese ions are shown as lilac spheres, the calcium ion as a green sphere, and waters and O atoms as small red spheres.

Photosynthesis is central to aerobic life and utilizes the CaMn_4_O_5_ centre of photosystem II (PSII) to split water and generate molecular oxygen through the four-step Kok cycle, harnessing the redox properties of manganese (Kok *et al.*, 1970 ▸). The definition of this catalytic centre and how it cycles through the Kok cycle to produce molecular oxygen has been a major goal in the field for over 30 years. The first crystal structure of PSII determined by SR crystallography appeared at the turn of the century at 3.8 Å resolution (Zouni *et al.*, 2001 ▸). This was improved to 1.9 Å resolution in 2011 using improved synchrotron beamlines, detectors and crystals (Umena *et al.*, 2011 ▸). Although these structures provided detailed insight into the organization of this 350 kDa multi-subunit complex, questions remained about the integrity of the CaMn_4_O_5_ centre in the dark stable state (S_1_) of PSII owing to X-ray-induced structural changes (Yano *et al.*, 2005 ▸, 2006 ▸). Shen and coworkers addressed this by femtosecond XFEL crystallography using the serial femtosecond rotational crystallography (SF-ROX) approach with cryogenically maintained large crystals of PSII (1.0 × 0.4 × 0.15 mm). By using more than a hundred such crystals, they were able to establish a damage-free structure of PSII at 1.95 Å resolution (Suga *et al.*, 2015 ▸). Most of the Mn–Mn distances were found to be shortened by 0.1–0.3 Å compared with the structure obtained using SR X-rays, with slight changes in the Mn–O and Mn–ligand distances. As in the SR structure, one of the oxygens, O5, of CaMn_4_O_5_ was found to be unusually distant from the nearby manganese ions, suggesting that O5 may participate in O=O bond formation. Using time-resolved serial femto­second crystallography (Suga *et al.*, 2017 ▸), the structural changes in PSII induced by two-flash illumination at room temperature have been defined, establishing the changes that occur between the S1 and S3 states. These heroic experiments provided structures to ∼2.35 Å resolution at room temperature and used over two million crystals of ∼20 µm in size. A water molecule located 3.5 Å from the Mn_4_CaO_5_ cluster disappeared from the map upon two-flash illumination, reducing the distance between another water molecule and the O4 atom and indicating a proton-transfer event. This was accompanied by the appearance of a positive peak around O5: a unique μ_4_-oxo bridge located in the quasi-centre of Mn1 and Mn4 (Fig. 2 ▸). This suggested the insertion of a new O atom (O6) close to O5, providing an O=O distance of 1.5 Å between these two O atoms consistent with the formation of an O=O bond.

### 2.2. Copper nitrite reductase in denitrification   

Copper nitrite reductases (CuNiRs) perform the first committed step of denitrification (NO_2_^−^ + e^−^ + 2H^+^ ↔ NO + H_2_O) and have been extensively studied over the last 25 years using SR crystallography. CuNiR was the first copper protein for which a structure was determined at atomic resolution (Ellis *et al.*, 2003 ▸), *i.e.* with a resolution of better than 1.2 Å, the resolution at which carbon–carbon bonds can be resolved. Denitrification is not only important from a bioenergetics perspective, but it is also crucial in terrestrial and oceanic nitrogen cycling and makes a significantly increasing contribution to global warming by the release of N_2_O, an ozone-depleting and greenhouse gas that is some 300-fold more potent than CO_2_.

**Figure 3:**
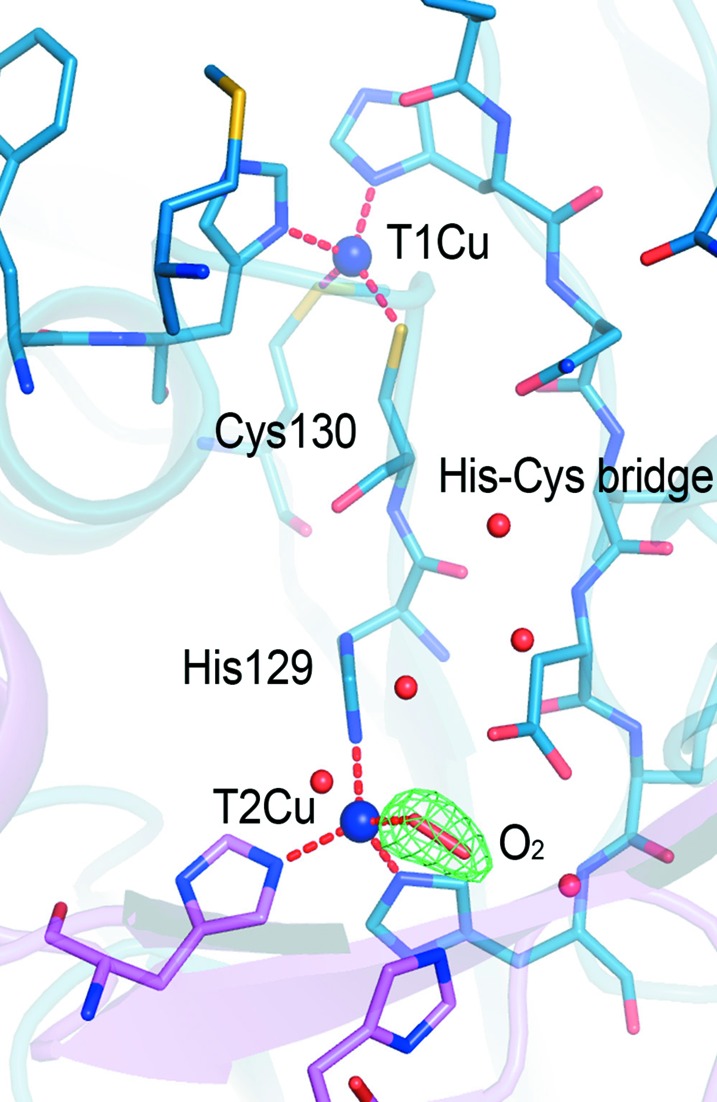
SF-ROX damage-free structure of nitrite reductase from *A. xylosoxidans* reveals a dioxygen species at the catalytic copper site. The OMIT *F*_o_ − *F*_c_ electron-density map is contoured at the 5σ level around the dioxygen molecule and is coloured green.

CuNiRs utilize two types of copper, T1Cu and T2Cu, where T1Cu receives an electron from a cognate partner while catalysis occurs at T2Cu through a displacement mechanism. The substrate replaces a water molecule before being converted to nitric oxide through well controlled and regulated proton and electron transfer. The two copper redox centres are linked together by a Cys130–His129 bridge, which is a novel feature of these enzymes, in which two redox centres are linked via neighbouring residues to form the catalytic cores. The T1Cu centres in these novel catalytic cores are prone to conversion to a reduced state by X-rays during a typical X-ray crystallographic data collection (Hough *et al.*, 2008 ▸). The T2Cu site of the resting-state enzyme, in contrast, remains unaffected at much higher X-ray doses. When the substrate nitrite is bound at T2Cu, it converts quickly to NO during X-ray data collection, indicating that electron transfer from T1Cu to T2Cu is regulated and occurs efficiently when substrate nitrite is present for catalysis. These features have been exploited to obtain a large number of structures from one crystal and to build up a structural movie capturing the conversion of nitrite to nitric oxide and its subsequent return to the resting state. This serial crystallography approach, termed multiple structures from one crystal (MSOX; Horrell *et al.*, 2016 ▸), has been made possible only recently by microfocus beams and efficient photon-counting detectors, minimizing the X-ray dose to a level enabling the determination of several structures from the same sample volume. This X-ray radiolysis approach to drive a chemical reaction is thus, in principle, applicable to all redox catalysts (Schlichting *et al.*, 2000 ▸). It is clear from the above discussion that it is most likely that none of the structures of CuNiRs collected at powerful synchrotron beamlines represent a ‘damage-free’ structure. SFX and SF-ROX XFEL crystallography have been used to study a number of CuNiRs [NiRs from *Alcaligenes faecalis* (*Af*NiR; Fukuda, Tse, Nakane *et al.*, 2016 ▸), *Geobacillus thermodenitrificans* (*Gt*NiR; Fukuda, Tse, Suzuki *et al.*, 2016 ▸) and *Alcaligenes xylosoxidans* (*Ax*NiR; Halsted *et al.*, 2018 ▸)] to obtain damage-free structures of the resting state as well as a number of catalytically important forms. In the case of *Ax*NiR, the resting state of *Ax*NiR obtained by SF-ROX using 64 large (1 × 0.8 × 0.05 mm) blue crystals unexpectedly revealed an O_2_ ligand bound to the T2Cu in a brand-new binding mode for a diatomic ligand in CuNiRs (Fig. 3 ▸). The observation of O_2_ in a time-frozen structure of the as-isolated oxidized enzyme provided long-awaited evidence for the mode of O_2_ binding in CuNiRs. This provided insights into how this CuNiR functions as an oxidase, reducing O_2_ to H_2_O_2_, or as a superoxide dismutase (SOD), since it was shown to have significant dismutase activity 20 years ago (Strange *et al.*, 1999 ▸). We note that a number of CuNiRs have been observed to function as oxidases, reducing O_2_ to H_2_O_2_, or even as superoxide dismutases (SODs), but this remains a relatively unexplored aspect of CuNiR catalysis.

### 2.3. Rhodopsins   

Rhodopsin is a G-protein-coupled receptor and is a light-driven proton pump that is central to vision by the human eye in dim light. Using very low dose EM, Henderson and Unwin determined the first (7 Å resolution) structure of bacterio­rhodopsin more than 40 years ago, showing the detailed arrangement of seven transmembrane α-helices (Henderson & Unwin, 1975 ▸). This work proved that integral membrane proteins have a tertiary folding with the same secondary-structure elements as water-soluble proteins. In the 1990s, Henderson and coworkers succeeded in obtaining the first atomic structure of bacteriorhodopsin to 3.5 Å resolution using electron diffraction from two-dimensional crystals (Henderson *et al.*, 1990 ▸; Subramaniam *et al.*, 1993 ▸). Synchrotron X-ray crystallography provided the structure to a resolution of 2.5 Å in 1997 using the microfocus beamline at the ESRF, which was the best beamline at the time to handle 20 × 20 × 5 µm crystals (Pebay-Peyroula *et al.*, 1997 ▸). The higher resolution improved the loop conformations and a number of side-chain residues. It also helped to identify eight water molecules constituting the proton-translocation pathway in the ground state. The first crystal structure of a mammalian rhodopsin, bovine rhodopsin, was obtained at 2.8 Å resolution at the turn of the century by the MAD method using data collected at SPring-8 (Palczewski *et al.*, 2000 ▸). Although close similarity was found to the structures of bacterial rhodopsin, the arrangement of the seven helices was found to be different, with larger and more organized extra-membrane regions than those in bacteriorhodopsins. During the last three years, damage-free structures of human rhodopsin and bacterio­rhodopsin have been determined using XFEL crystallography at SACLA and LCLS. In an attempt to define the structural changes that accompany the photocycle of bacteriorhodopsin proposed over half a century ago, a pump–probe approach has been used to construct a structural movie at ∼2 Å resolution, maintaining the microcrystals at 20°C. Nearly two million crystals were used in each of two independent studies: one carried out at SACLA using a nanosecond pump laser and the other at LCLS using a femtosecond pump laser (Nango *et al.*, 2016 ▸; Nogly *et al.*, 2018 ▸). In the SACLA experiment, a set of 13 time delays between the pump laser pulse and the XFEL pulse were chosen to build a structural movie: Δ*t* = 16 ns, 40 ns, 110 ns, 290 ns, 760 ns, 2 µs, 5.25 µs, 13.8 µs, 36.2 µs, 95.2 µs, 250 µs, 657 µs and 1.725 ms. The SACLA experiments were complemented by those at the LCLS, where different sets of time delays between the pump laser pulse and the XFEL pulse were chosen (300 fs, 600 fs, 900 fs, 1100 fs and 10 ps) to capture the isomerization of retinal at 1.5 Å resolution. These experiments showed how excited all-*trans* retinal samples different conformational states within the binding pocket of the protein before passing through a twisted geometry and emerging in the 13-*cis* conformation.

## 3. CryoEM: some recent highlights providing atomic details of complex structures   

The ability of cryoEM to determine structures that have proven intractable to other methods to sub-4 Å resolution has provided a series of high-profile structures over the last few years. The transformation of cryoEM into the ‘method of the decade’ has come from steady progress over the last 20 years on many technical fronts. These include developments in direct electron detectors, improved microscope design and more sophisticated data-processing algorithms (Subramaniam *et al.*, 2016 ▸). Many important cellular processes are governed by complex protein assemblies. The ribosome, the determination of the structure and mechanism of assembly of which by SR X-ray crystallography was recognized by a Nobel prize in 2009, has long been used in electron-microscopy experiments (Frank & Agrawal, 2000 ▸; Lata *et al.*, 1996 ▸); indeed, ∼760 entries in the EMDB relate to the ribosome. Recent years have seen not only an improvement in the resolution of these complexes by EM, but also an increase in the diversity of the systems studied. The recent structures of the spliceosome, ubiquitination and the respirasome are some examples. Here, we briefly discuss two cases.

### 3.1. Respirasome   

**Figure 4:**
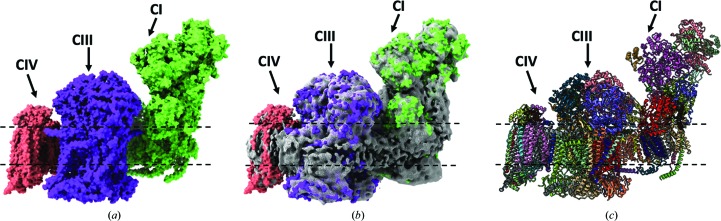
Structure of the respirasome determined to 5.4 Å resolution by single-particle cryoEM (Gu *et al.*, 2016 ▸). The modelled structure is shown as a surface view (*a*), fitted within the EM map (*b*) and as a ribbon diagram (*c*). The three main components, complex I (CI), complex III (CIII) and complex IV (CIV), are labelled along with the bilayer (dashed lines).

The development of our structural understanding of the respiratory chain has been advanced by new cryoEM structures. For example, new understanding has been reached as to how the proton gradient is harnessed by ATP synthase to produce ATP, with a surprising organization of the main proton-translocation domain (Kühlbrandt & Davies, 2016 ▸). Removing the need for crystallization, which can offer significant challenges for larger protein complexes, has permitted the solution of not just large protein complexes in the respiratory chain such as ATP synthase (Zhou *et al.*, 2015 ▸), cytochrome *bc*_1_ (Amporndanai *et al.*, 2018 ▸) and complex I (Agip *et al.*, 2018 ▸), but also supercomplexes such as the 1.7 MDa respirasome, which offers insights into the arrangement of respiratory-chain complexes in the mitochondria. Four respirasome structures have been published to date from porcine, ovine and bovine sources, all revealing a similar architecture consisting of a complex I core with a complex III dimer (cytochrome *bc*_1_) packed against the side, with the complex III dimer packed between the complex I core and complex IV (Fig. 4 ▸; Gu *et al.*, 2016 ▸; Wu *et al.*, 2016 ▸; Letts *et al.*, 2016 ▸; Sousa *et al.*, 2016 ▸). The interaction of complex I and complex III is facilitated by NDUFA11, which directly interacts with both subunits, acting to bridge the gap between the two subunits and ensure close packing interactions. Moreover, it has been shown that three lipids are also involved in this interaction and may behave like a ‘glue’ to propagate protein–protein interactions. The supercomplexes form the basis of new models to understand how electron transfer is coordinated between the subunits. It is interesting to note that higher molecular-weight species have been detected, and it will be interesting to see, using developments such as electron tomography, how complex formation occurs within the bilayer environment as opposed to detergent-extracted particles. This is an example where combining high-resolution X-ray structures of individual components and an ∼5 Å resolution EM structure of the whole supercomplex machinery could prove to be a powerful toolset to open new ways of looking at the biological workings at higher levels.

### 3.2. Proteins involved in neurodegenerative diseases   

Amyloid plaques, fibre formation and the aggregation of proteins are signatures of a number of neurodegenerative diseases. It has been shown that these are formed from life-sustaining abundant proteins that become defective, described in the field as ‘gain of function’. Our understanding of several important neurodegenerative diseases has rapidly changed recently with a series of landmark papers detailing the structure of tau and amyloid-β proteins (Fitzpatrick *et al.*, 2017 ▸; Gremer *et al.*, 2017 ▸). These new structures provide unprecedented detail into the structural arrangement of this important clade of proteins. Interestingly, for tau different distinct folds can be seen in Pick’s disease and Alzheimer’s disease, despite the same building blocks, with phosphorylation predicted to play a role (Falcon *et al.*, 2018 ▸).

## 4. Structure-guided drug discovery   

Crystallographic structure-based drug development has yielded several notable drugs during the last 30 years. In the 1990s several drugs emerged from structure-guided approaches, including Trusopt (targeting carbonic anhydrase for glaucoma), Agenerase, Aluviran and Viracept (AIDS drugs targeting HIV protease), and Relenza and Tamiflu (influenza drugs developed using the crystal structure of neuraminidase), thus establishing crystallographic structural information as an essential ingredient for most major drug-discovery programs. Structure-based or structure-guided drug design often occurs in a cycle, in which new molecules are synthesized, tested and crystallized with the target protein. The structural analysis helps to evaluate the binding mode and suggests ways to modify the molecule to either enhance the binding or prevent off-target binding. In the last 20 years, fragment-based drug discovery (Shuker *et al.*, 1996 ▸) has made a significant impact as an approach that is able to cover a large amount of chemical space, which, when combined with structural information, can rapidly lead to candidate drug molecules. Notable examples (Blundell, 2017 ▸) of successful fragment-derived drugs are vemurafenib (which targets a mutant form of BRAF, a kinase, thus extending life for patients with skin cancer), venetoclax^1^ (which binds to BCL-2 to treat chronic lymphocytic leukaemia) and ribociclib, which was developed by Astex and Novartis to target the protein kinase Cdk4 as a first-line treatment for advanced breast cancer in combination with letrozole.

### 4.1. The role of cryoEM in drug discovery   

During the last five years, cryoEM has become a powerful approach in structure determination, particularly of membrane proteins or large complexes. Although still limited in resolution compared with both X-ray crystallography and NMR, it can provide valuable insights into inhibitor binding. For example, within the transient receptor potential channel (TRP) membrane-protein family there had been a paucity of structural information on the full channels, with most structural work using the ‘divide-and-conquer’ approach of crystallizing the more amenable soluble domains. However, since 2013, ∼50 new single-particle cryoEM structures have been determined from all the major clades (TRPM, TRPA, TRPV and TRPC; Madej & Ziegler, 2018 ▸). A common feature of these channels has been the presence of bound inhibitors, which have often been used to stabilize and ‘lock’ the target to improve the resulting resolution. For TRPV1 not only was the binding site for the spider toxin identified, but the role of lipids in facilitating this interaction could also be seen for the first time by using nanodisc technology (Gao *et al.*, 2016 ▸). Another example is the case of imidazoleglycerolphosphate-dehydratase (IGPD), an essential enzyme in histidine biosynthesis. The *Arabadopsis* homologue was highly amenable to crystallization, resulting in several high-resolution structures (Bisson *et al.*, 2016 ▸). These structures not only revealed the mode of binding, but also the molecular basis for the nanomolar equipotency of potent enantiomers. However, the structure of the yeast isoform, which is more sensitive to small-molecule inhibitors and intractable to crystallization, has recently been determined by cryoEM. This revealed stabilization of the inhibitor-binding loop in the yeast homologue, ‘locking’ the inhibitor in the pocket, which was predicted to create a higher sensitivity to triazole-phosphonate inhibitors in the yeast homologue compared with that from *Arabidopsis* (Rawson *et al.*, 2018 ▸).

For some medically important systems, a significant hurdle in structure determination is obtaining sufficient quantities of material. This can often be a limiting factor, despite the development of crystallization robotics that can dispense nanolitre droplets of protein sample. The large amount of screening space that is required to find a suitable crystallization condition can be a limiting factor. For large protein complexes this becomes more challenging, and specialized cellular machinery may be required for the synthesis of sufficient protein for extraction from host tissue. In contrast, EM is less demanding on the quantity of sample than a typical X-ray or NMR experiment. This is highlighted by the structure of the malarial translocon, which is essential for exporting effector proteins over the membrane (Ho *et al.*, 2018 ▸). The recent cryoEM structure to 3.5 Å resolution will provide new avenues for structure-based drug-design pipelines. Through substrate profiling, inhibitors have been designed based on structural differences between the human and plasmodium 20S proteasome, with the binding of these inhibitors shown by cryoEM. This impressive study shows the power of cryoEM when tackling proteins from a native source (Li *et al.*, 2016 ▸).

### 4.2. Antimalarials targeting cytochrome *bc*_1_   

One example where X-ray crystallography and cryoEM have been used in a complementary and powerful manner is for the antimalarial target complex III or cytochrome *bc*_1_. According to data from the World Health Organization, the infection rate of malaria has declined by 41% since the turn of the century, but 212 million new cases and 429 000 deaths still occurred globally in 2015. Targeting the electron-transport chain (ETC) of plasmodial mitochondria has been shown to be therapeutically successful. Atovaquone, an inhibitor of the cytochrome *bc*_1_ complex, used in conjunction with proguanil (the drug combination Malarone) remains in clinical use in its generic formulation after the expiry of the GSK patent in 2013 after usage for 14 years. Increasing resistance and the expiry of the patent resulted in extensive research to find alternative drugs that target the ETC in general and cytochrome *bc*_1_ in particular.

**Figure 5:**
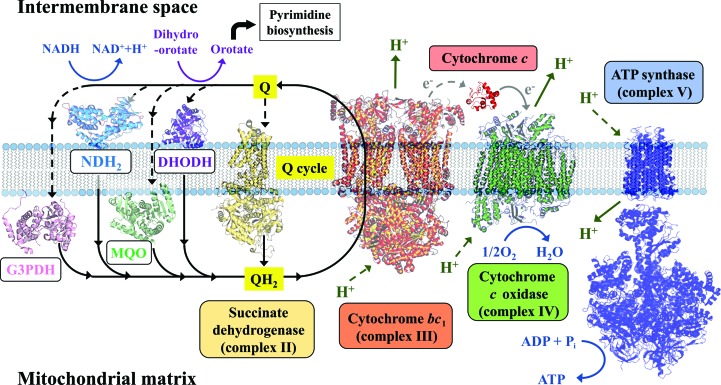
The plasmodial mitochondrial electron-transport chain.

**Figure 6:**
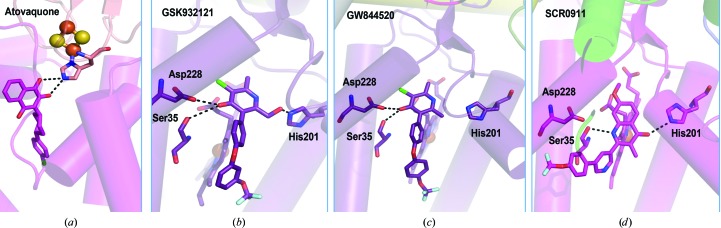
Q_o_ and Q_i_ sites of cytochrome *bc*_1_, with inhibitors bound in the cytochrome *b* subunit. (*a*) *S. cerevisiae* structure with atovaquone bound in the Q_o_ site, making a hydrogen bond to the His121 side chain from the Rieske iron–sulfur protein. S and Fe atoms are shown as yellow and orange spheres, respectively (Birth *et al.*, 2014 ▸). (*b*, *c*, *d*) Bovine cytochrome *bc*_1_ with (*b*) GSK932121, (*c*) GW844520 (Capper *et al.*, 2015 ▸) and (*d*) SCR0911 inhibitors bound in the Q_i_ site (Amporndanai *et al.*, 2018 ▸). Residues and inhibitors are shown as sticks. Selected possible hydrogen bonds are illustrated by black dashed lines.

Cytochrome *bc*_1_ exists as a heterodimer of two multi-subunit proteins embedded in the inner mitochondrial membrane. It facilitates electron transport to cytochrome *c* via the Q cycle, in which electrons are utilized at two sites of the cytochrome *b* subunit for the oxidation of ubiquinol (at the Q_o_ site) and the reduction of ubiquinone (at the Q_i_ site) (Fig. 5 ▸). The subunit composition of cytochrome *bc*_1_ can vary between species. Prokaryotic complexes only contain 3–4 subunits, while mitochondrial proteins contain 10–11 different subunits (Trumpower, 1990 ▸). However, all cytochrome *bc*_1_ complexes have a catalytic core containing three essential subunits: cytochrome *b*, cytochrome *c*_1_ and the Rieske iron–sulfur protein. In the absence of the structure of plasmodial cytochrome *bc*_1_, the structure of *Saccharomyces cerevisiae* cytochrome *bc*_1_ has been used as a surrogate for that of the parasite, showing atovaquone in the Q_o_ site (Birth *et al.*, 2014 ▸), where its location is enforced by a polar contact between the O atom (O3) of the hydroxyl group of the ligand and the His181 side chain from the Rieske protein (Fig. 6 ▸). The inhibitor binding explained the broad target spectrum, species-specific efficacies and acquired resistances (Stickles *et al.*, 2015 ▸).

**Figure 7:**
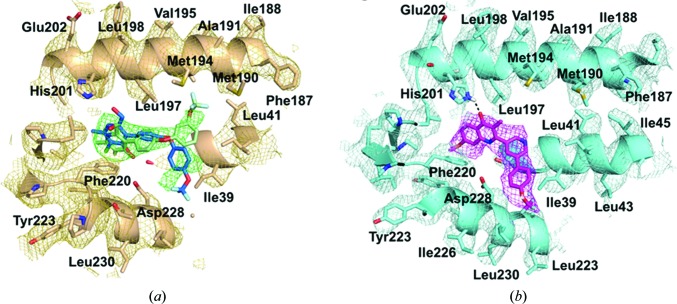
The Q_i_ site of bovine cytochrome *bc*_1_ in a cryoEM map with antimalarial compounds (Amporndanai *et al.*, 2018 ▸). (*a*) The Q_i_ site with GSK932121 density (coloured green) suggests there are two modes of inhibitor binding accompanied by rotation around the oxygen–carbon bond. The binding pose shown in green agrees with the crystal structure, with the trifluoromethyl group pointing towards Met194. There is additional density which suggests that the trifluoromethoxy­phenyl group could be rotated and point towards Asp228, revealing an additional mode of binding (shown in blue). (*b*) The Q_i_ site with the inhibitor SCR0911 (shown in pink) located in strong density. A possible hydrogen bond from the inhibitor to His201 is shown as dashed line and is clearly defined in EM density. For all maps, the density is contoured at 3σ.

Crystallographic studies have been important in suggesting the development of dual-site inhibition of cytochrome *bc*_1_ as a valuable strategy for antimalarial combination therapy, one of which is combining atovaquone with ELQ-300 targeting the Q_i_ site of cytochrome *b* (Stickles *et al.*, 2015 ▸). The discovery of a connection between the cardiotoxicity of the 4(1*H*)-pyridone class of inhibitors, GSK932121 and GW844520, and the ability of this class to overcome parasite Q_o_-based atovaquone resistance has provided new opportunities. These compounds bind to the Q_i_ site of cytochrome *b* in bovine cytochrome *bc*_1_, a surrogate of the host (Capper *et al.*, 2015 ▸), encouraging the development of Q_i_ binders [Figs. 6 ▸(*b*), 6 ▸(*c*) and 6 ▸(*d*)] with the goal of decreasing the cardiotoxicity of the compounds and developing stronger inhibitors for plasmodial cytochrome *bc*_1_. Crystal structures show that the carbonyl O atoms of the ligand are within 3.5 Å of both Ser35 in loop A and OD1 of Asp228 in loop E, allowing the formation of possible hydrogen bonds. All recently structurally identified Q_i_ binders, including SCR0911 (Amporndanai *et al.*, 2018 ▸) [Fig. 6 ▸(*d*)], make similar potential hydrogen bonds within the Q_i_ site to either His201, Ser35 or Asp228 independently or a combination of these residues. It has been shown that small changes in the 4(1*H*)-quinolone scaffold can change the binding site from Q_i_ to Q_o_, which makes it crucial that these are visualized directly in experimentally determined structures. An alternative approach for proving the exact binding site is to test the drug action against a *Plasmodium falciparum* strain containing the Q_i_ site mutation I122L in cytochrome *b*. However, a significant limitation of the crystallographic approach is the requirement for significant quantities of protein, with concentrations of ∼50 mg ml^−1^ being required for crystallization. This reliance on protein and an inability to overexpress it limits studies of the malarial homologue itself. Therefore, a cryoEM approach, although limited in resolution, could provide a link by being suitable for the study of systems in which protein is more limiting. As a proof of principle, the structure of the bovine cytochrome *bc*_1_ complex was recently determined by single-particle cryoEM to ∼4.0 Å resolution with bound inhibitors (Fig. 7 ▸), which were clearly identified within the Q_i_ site (Amporndanai *et al.*, 2018 ▸). This opens up the potential for using a similar approach to study the plasmodial cytochrome *bc*_1_ and other complexes of the mitochondrial ETC for both enzyme-mechanism studies and structure-guided development of antimalarial drugs. Moreover, recent cryoEM studies have now revealed the structure of cytochrome *bc*_1_ from *Flavobacterium johnsoniae*, showing a unique arrangement of the complex (Sun *et al.*, 2018 ▸).

## 5. Ease of access, data quality and appraisal of structures from cryoEM and X-ray crystallography   

A significant limiting factor in the cryoEM field with respect to structure determination is the availability of high-end instrumentation which is open to ‘non-expert’ users. The high capital cost of the microscopes and the continued operating and maintenance cost with appropriate technical staff is a significant undertaking for a university-based group. Despite this, new facilities are being built across the UK and internationally which have opened their doors to ‘non-expert’ users, thus contributing to the expansion of the community. In addition, national facilities are emerging, some of which are co-located with synchrotron facilities, for example eBIC at the Diamond Light Source (Saibil *et al.*, 2015 ▸). The success of these is reliant upon sufficient training and the availability of lower specification microscopes for screening at the institutional/regional level that are well supported. Furthermore, the determination of new EM structures requires typical data-collection times of the order of days for single-particle EM projects, compared with minutes for X-ray crystallographic data. Screening of samples is also more challenging in cryoEM. Even with ‘on-the-fly’ data processing, a sufficient amount of data must be collected before one can obtain a good estimation of factors such as homogeneity, orientation distribution and particle number per micrograph, which all play a role in the final resolution. In contrast, screening crystals can be much more rapid. A single test image, even at the home source, gives a good approximation of the final resolution at a synchrotron, albeit with radiation damage and anisotropy during data collection sometimes limiting the final resolution.

Although typical in crystallization experiments, the screening of conditions such as pH and salt concentration is often poorly defined in EM. The ability to quickly screen hundreds of crystals in a few hours from distinctly different conditions allows a broad chemical space to be quickly explored. This is not true for cryoEM experiments, where the ‘final resolution’ of a structure is only obtained after extensive image processing. Despite technological improvements in grid making, the quality of grids and sample uniformity remain a significant limiting factor. The automation of crystallographic facilities allows the selection of a well diffracting crystal for high-resolution data collection which can be processed ‘on-the-fly’, and in favourable cases the structure can be solved before the end of the session. In the case of SFX-based crystallography, two approaches for obtaining crystallographic data have emerged. One relies on a sample-injection system that requires small (∼2–20 µm) well diffracting crystals, while the other is a fixed-target rotational crystallographic (SF-ROX) approach that requires larger (>300 µm) crystals. Both of these approaches provide ‘damage-free’ structures owing to the intrinsic properties of the X-ray laser pulse, which is shorter in duration (∼10 fs) than atomic movement. The main limitations that remain are the availability of XFEL beamtime and the preparation of suitable samples and their delivery to the XFEL beams. Both EM and XFEL experiments are data-intensive and thus require large data-processing/storage capabilities.

There is also significant work to be performed in the validation of EM-determined structures. At worst, the use of high-resolution search models can significantly bias the end result (Henderson, 2013 ▸), and as with any modest resolution structure care must be taken when interpreting the resulting density (Neumann *et al.*, 2018 ▸). At the typical resolution of current cryoEM maps it can still be difficult to unambiguously assign small-molecule density and side chains. Robust validation software developed by the X-ray crystallographic community for analysing the resulting structural geometry (Ramachandran plot, clashscores, density-fit analysis) is available and it is important that this is routinely used when reporting EM structures. The lack of a conventional ‘*R* factor’ removes a useful metric for the quality of the model fit to the data. However, with the availability of phase information the maps generated by single-particle EM are not influenced by the refined structure, removing the ‘phase bias’ that may exist in some crystal structures. As in all structural biology, the key is in rigorous assessment of the data and making sure that the conclusions drawn are consistent with the resolution obtained. Moreover, showing both the map and model in the published structure rather than only the refined model allows the reader to make their own assessment of the strength of the conclusions drawn. This is even more important in the case of small-molecule binding, where in the reported structure the corresponding density may be absent or only be shown for the bound inhibitor and not the surrounding residues, making it difficult to assess the noise level within the binding site.

## 6. Concluding remarks   

The above case studies have demonstrated that the richness of structural biology can best be harnessed by combining methods where practical and possible. It is wonderful that structures can be obtained without crystallizing the biological system by using single-particle imaging by cryoEM, which indeed is proving to be the ‘method of the decade’. Structural details that are only available at atomic resolution (when atoms making C—C bonds are clearly resolved) are often the underpinning aspects of the chemistry that defines the mechanism of an enzyme or processes such as electron transfer, bond formation and breakage. Resolving structures at such atomic resolutions is going to continue to require well diffracting crystals, powerful synchrotrons and efficient detectors for the foreseeable future. The challenge for synchrotron-based crystallography is to minimize X-ray-induced damage, particularly when radiolysis can cause chemical changes. XFEL-based crystallography, by virtue of the nature of the source (femtosecond pulses), will continue to offer the unprecedented advantage of ‘damage-free’ structures at high resolutions at both ambient and cryogenic temperatures. It also offers the unique capability for time-resolved studies either via pump–probe experiments (Suga *et al.*, 2017 ▸; Nogly *et al.*, 2018 ▸) or by combining SFX with mix-and-inject systems (Stagno *et al.*, 2017 ▸). The impact of cryoEM will continue to expand and will remain critical in providing structural details of large macromolecular complexes. It is not inconceivable that the atomic details of the whole mitochondrial electron-transport chains of a number of important parasites will be resolved by cryoEM within the next five years. In the coming years, we can expect extensive efforts to be devoted to the rigorous validation of cryoEM structures, automatic building/refinement of models and opening of the method to the wider biological community, in a manner similar to X-ray crystallo­graphy. Further exciting developments are being seen in the field of MicroED (electron diffraction). The structure of lysozyme showed the power of this technique for studying protein structures, and recent work by the Gonen group has shown this to be a powerful approach for determining structures from very small crystals (Shi *et al.*, 2016 ▸). Two separate reports of the model system apoferritin solved to ∼1.6 Å resolution by single-particle cryoEM show the potential for gaining ‘high’ resolution EM data, although less robust systems will certainly require further development to reach such resolutions.

It is our view that the strength of structural biology going forward is likely to be in the combination of techniques, rather than in a sole technique expanding and dominating the field. Even with recent developments in EM, sub-65 kDa molecular masses remain a limiting factor, with the poor signal to noise in the raw images making particle identification and alignment a significant challenge. In crystallography, multi-protein complexes, particularly those which involve membrane proteins or where the complexes are not stable over the crystallization time scale, will remain a limitation. The serial crystallography approach developed at XFELs is likely to be implemented at the more powerful synchrotrons, many of which will undergo major upgrades in the next eight years. The new beamlines and detector technology, with remote data collections and robotic crystal mounting, offer significant gains in the speed and ease of synchrotron data collection. On-the-fly data processing allows the data quality to be quickly assessed and ensures that full high-quality data sets are collected.

Structural biology is commonly an ‘averaging’ technique, be that in the need for a homogeneous crystal with multiple repeating units or the requirement for tens of thousands of particles that must be averaged to improve the signal to noise in EM. One limitation of this approach is that it can lead to one or only a handful of states being determined, often those that dominate in the ground state. Capturing the structural changes that occur during catalysis or transport is a significant challenge, not least owing to the time scales of the events. However, recent years have seen a growing development of time-resolved methodologies. Landmark studies have shown that XFEL sources can provide femtosecond-to-millisecond information on biological processes, including catalysis (Johansson *et al.*, 2017 ▸; Stagno *et al.*, 2017 ▸). For larger conformational changes in the microsecond-to-millisecond time frame, time-resolved cryoEM can synergize with other methods such as SAXS, but can additionally provide atomic-level information (Frank, 2017 ▸). It should be noted that rapid developments in the field of mass spectrometry may provide an excellent partner for traditional structural techniques, and using fast photochemical oxidation of proteins (FPOP)-based approaches one can map structural changes in a time-dependent manner (Li *et al.*, 2018 ▸). Moreover, mass spectrometry can sort conformational heterogeneities and ‘soft-land’ these onto EM grids to overcome problems of sample heterogeneity. Although still some way from its full potential, it highlights the bright future in combining techniques (Benesch *et al.*, 2010 ▸).

Thus, the expanding toolkit of structural biology, with further advances in the technologies associated with cryoEM, synchrotron radiation and XFEL crystallography and their ease of use, will continue to enable the wider community to address more complicated and demanding scientific questions, thus ensuring the pole position of structural biology.

## Footnotes

1 According to *Drugs to Watch*, the forecast sale for venetoclax is 1.477 billion US$ (http://images.info.science.thomsonreuters.biz/Web/ThomsonReutersScience/%7B7e677448-1313-40ae-9978-6be1a8532a08%7D_A4_MarketInsight_Report_0213_012__edits_2.pdf).
